# Knowledge assessment on cleft lip and palate among recently graduated dentists: a cross-sectional study

**DOI:** 10.1186/s12903-023-03388-y

**Published:** 2023-09-25

**Authors:** Bahn Agha, Narmin Mohammed Saeed Helal, Thaer Jaber Al-Khafaji, Ghada Abdullah Farie, Osama Basri, Padhraig S. Fleming

**Affiliations:** 1 Department of Pedodontics, Orthodontics and Preventive Dentistry, College of Dentistry, Mustansiriyah University, Baghdad, Iraq; 2 Pediatric Dentistry Department, Faculty of Dentistry, King Abdulaziz University, Jeddah, Saudi Arabia; 3 Pedodontics, Orthodontics and Preventive Dentistry Department, College of Dentistry, University of Babylon, Babylon, Iraq; 4 Department of Dentistry, King Faisal Specialist Hospital and Research Center, Jeddah, Saudi Arabia; 5 Dublin Dental University Hospital, The University of Dublin, Trinity College Dublin, Dublin, Ireland

**Keywords:** Nonsyndromic clefting, Congenital defects, Dental internship, Questionnaire, Curriculum

## Abstract

**Background:**

The complex presentation, associated co-morbidities and multi-disciplinary requirements dictate the requirement for in-depth knowledge in order to effectively manage patients with cleft lip and palate (CLP). We aimed to develop a validated questionnaire for cleft lip and palate knowledge assessment and to evaluate the knowledge of cleft lip and palate among a group of recently-graduated dentists.

**Materials and methods:**

A multiple-site, cross-sectional questionnaire-based study was conducted. The study population included recently graduated dentists involved in a dental internship program. A bespoke questionnaire was developed and validated, with internal consistency assessed using Cronbach’s alpha and factor analysis performed. A 47-item prototype was distilled into a 15-item questionnaire. This was distributed to the participants with a response rate of 67% obtained.

**Results:**

The overall proportion of correct responses among dental interns was moderate (73%). The best results were found in relation to CLP treatment including the effect of unfavorable surgical outcomes on speech (89.5%) and the impact of CLP on the occlusion (87.6%). The lowest rate of correct responses (26.7%) was identified in relation to the association between CLP and smoking.

**Conclusion:**

A validated CLP questionnaire was developed, permitting evaluation of the knowledge of cleft lip and palate and its management among recently graduated dentists. There is limited appreciation among dental interns of the risk factors for CLP as well as post-surgical complications. Given that general dentists are often the gatekeepers for the management of patients with cleft lip and palate, it is important that the findings of this survey are used to inform the curriculum and teaching of cleft lip and palate.

**Supplementary Information:**

The online version contains supplementary material available at 10.1186/s12903-023-03388-y.

## Background

Cleft lip and palate (CLP) is one of the most prominent hereditary diseases affecting newborns. Clefts occur in the early stages of human embryonic development and are categorized as “non-syndromic” if the malformation appears to be an isolated defect or “syndromic” if the malformation is a part of a larger disorder in a known pathologic pattern. The former represents approximately 70% of facial congenital malformations [1].

The etiology of CLP is thought to be multifactorial, resulting from a combination of genetic and environmental factors [2]. Advanced maternal age, smoking, alcohol consumption, and deficiency in folic acid and B6 and B12 vitamins during pregnancy are associated with an increased risk of CLP [3, 4].

A number of genes and molecular pathways have been linked to the etiology of clefting. An understanding of the molecular mechanisms of cleft formation is therefore important in supporting decision-making and counselling [5].

The prevalence of CLP varies according to race, geographic location, environmental exposure, and social and economic conditions with the highest prevalence found among Asians and Native Americans (1/500), while the lowest prevalence observed among Africans (1/2500). Caucasians have an intermediate prevalence of 1 in 1,000 [2, 4].

With respect to gender, the prevalence of CLP is approximately double that of females [6, 7]. In addition, blood-related couples are at significantly greater risk of having children with congenital defects and genetic disorders [8]. Based on a recent meta-analysis, the global prevalence of cleft palate (CP), cleft lip (CL), and CLP in every 1000 live births was 0.33, 0.3, and 0.45, respectively [9].

A multidisciplinary team strategy is essential to handle patients born with cleft lip and palate conditions [10]. Although every patient’s path is different, many patients with the same cleft phenotype go through similar pathways, including maxillofacial, auditory, speech and language, psychology, pediatric, restorative, and orthodontic clinics. However, dentistry remains essential to several aspects of cleft treatment [11].

In view of the disparate nature of the condition and associated co-morbidity, the adequate provision of dental services to patients with CLP can be challenging [10]. These challenges are exemplified by a failure to identify improved dental outcomes in the recent Cleft Care UK study relative to the findings of the Clinical Standards Advisory Group [12]. Conversely, a general enhancement in psychological, surgical, facial proportions, and speech and language outcomes were noted [13–15].

From a global perspective, Mossey [16] highlighted the universal challenges in the orofacial cleft field, such as the absence of awareness, the failure to differentiate between orofacial cleft sub-phenotypes, and the lack of standardization of cleft classification. Primary prevention of non-syndromic clefts involves intensive research into the genetic and environmental factors implicated in the etiology. Additionally, prevention is applied to the management of the consequences of being born with a cleft, such as dental caries, malocclusion, and psychosocial adjustment [16].

Although several educational guides for cleft lip and palate conditions are available in the literature [17], there is still a shortage of CLP knowledge among dental students and/or recently graduated dentists [18]. In addition to orthodontists and pediatric dentists, general dentists often play a pivotal role in the diagnosis and management of patients with cleft lip and palate. Therefore, it is important that dental students have an understanding of the diagnosis and management of CLP. In particular, it is recognized that early detection, referral, and diagnosis of children with CLP improves both the treatment prognosis and quality of life for both parents and children [19, 20].

Knowledge assessment is an essential tool for improving the performance of dental students and healthcare providers [21]. Several knowledge evaluation tools are available, such as survey questionnaires, faculty self-reported assessment survey, scale assessment tools, and knowledge maps; however, a questionnaire survey is particularly suited to providing evidence of practice and knowledge [22]. To apply this tool in real-life practices, validity, reducibility, and adequate response rates with minimum bias are prerequisites [23].

In this study, we aimed to develop a validated questionnaire for CLP knowledge assessment and to probe the current knowledge of CLP among a cohort of recently graduated dentists in order to better inform the teaching of CLP within undergraduate dental education.

## Materials and methods

### Study design and setting

Ethical approval was obtained from the King Abdulaziz University Research Ethics Committee (REC D/4/98,976). Informed consent was obtained using an electronic form. A questionnaire-based cross-sectional study was conducted in Jeddah, Saudi Arabia. Over a four-month period, 156 recently graduated dentists were invited to participate, though only 105 complete responses were obtained. The study population includes recently graduated dentists enrolled in a dental internship program in Jeddah. The age range was between 23 and 30 years, with a male preponderance (Table 1).

**Table 1:** Demographic data and questionnaire results

Demographic data Total n = 105	Sample n (%)	Number of correct responses (0–15 questions) Mean (± SD)	P value
Gender			
Male	57 (54.3)	10.3 (2.4)	< 0.01**
Female	48 (45.7)	11.8 (2.6)	
Age (years)			
23–25	71 (67.6)	10.9 (2.9)	0.63
26–28	29 (27.6)	11.0 (1.7)	
29–30	5 (4.8)	11.8 (0.8)	
Grade Point Average (GPA)			
A	41 (39.0)	11.6 (2.2)	0.07
B	44 (41.9)	10.8 (2.8)	
C/D	20 (19.0)	10.1 (2.5)	
Affiliation			
King Abdulaziz University	55 (52.4)	10.8 (2.9)	0.37
Al-Farabi Private College	16 (15.2)	10.9 (2.7)	
Ibn Sina National College	5 (4.8)	12.6 (0.9)	
OthersϮ	29 (27.7)	11.0 (2.1)	

n, number of the participants, * Significant (P < 0.05); ** Highly Significant (P < 0.01); Ϯ Umm Al-Qura University, Batterjee Medical College; SD, Standard Deviation

### Questionnaire and procedure

The questionnaire was divided into two main parts: demographic data and 47 questions incorporating four categories: (1) General knowledge of CLP; (2) Early interventions for CLP; (3) Interdisciplinary care; and (4) Management of CLP. Pre-defined responses were included as follows: “Agree or Yes,” “Disagree or No,” and “I don’t know” (Supplementary File 1). The questionnaire was subjected to validity and reliability assessment tests, with the correct answers informed by subject experts [24].

The consent form and the validated version of the questionnaire (Supplementary File 2) were distributed electronically to the recently graduated dentists who attended the internship program. Subsequently, the anonymous responses were collected and analyzed by the investigators (BA, NH, and PF).

### Validity and reliability

**Fig. 1:**
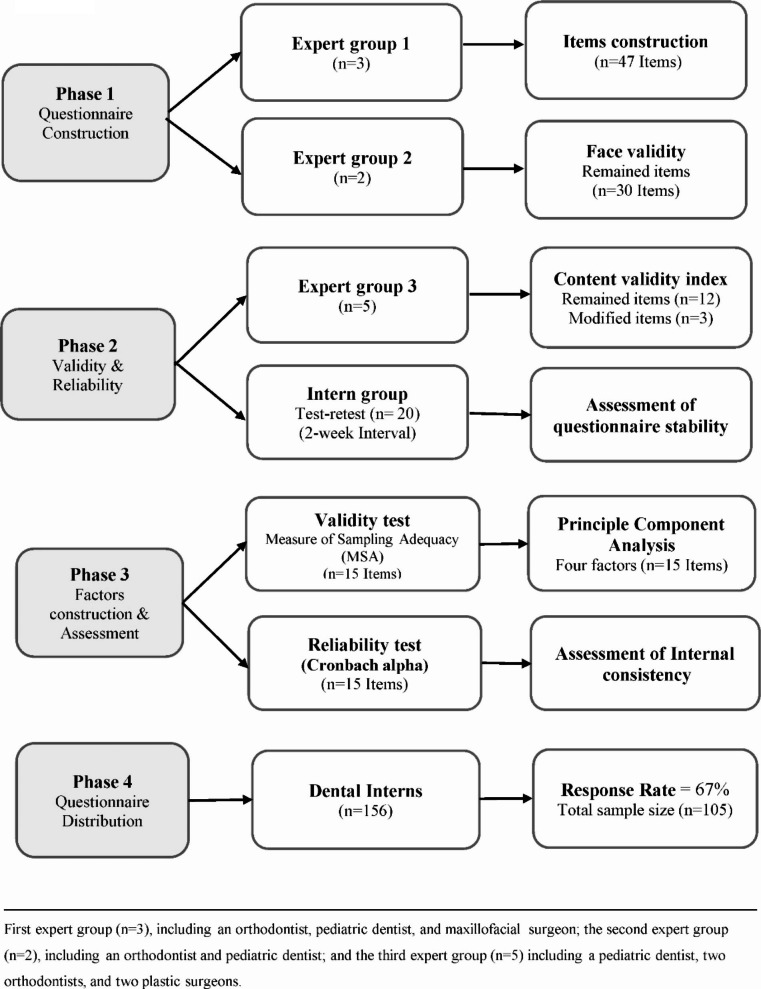
Flowchart demonstrating the faces of questionnaire development

The preliminary questionnaire, consisting of 47 questions (Supplementary File 1), was created by the first expert group (n = 3). Consequently, the second expert group (n = 2) re-examined the questionnaire to confirm its face validity. The questionnaire was therefore refined to include 30 questions (Fig. 1). Subsequently, the 30-item questionnaire was independently evaluated by a third expert group (n = 5).

Content validity was evaluated based on four domains (relevance, clarity, simplicity, and ambiguity). The scoring for the questions was as follows: 1 = not relevant, 2 = somehow relevant, 3 = quite relevant, and 4 = highly relevant. Any question that scored 1 or 2 was excluded [24]. According to Lynn’s criteria [25], excellent content validity, with five or fewer experts, is achieved when the item content validity index (I-CVI) is scored one, which means all experts must agree on the validity of the content [24]. Thus, any item with an I-CVI of less than one was excluded. After validity assessment, 12 out of 15 questions were retained and unaltered, while the other three were modified. Finally, a 15-item questionnaire was developed (Supplementary File 2).

The reliability of the questionnaire was explored using the *Test-Retest* approach to measure the stability of the questionnaire items, with 20 participants answering the questionnaire twice within 2-week intervals. Kappa values ranged between 0.6 and 0.8, indicating substantial agreement. *Cronbach’s alpha* was used to evaluate the internal consistency of the questionnaire items.

### Principal component analysis

The structure of the 15-item questionnaire was evaluated via factor analysis (Principal component and Varimax rotation) with sufficient inter-correlations among questionnaire items and a Kaiser-Meyer-Olkin Measure of Sampling Adequacy (MSA) of 0.67 to determine the possibility of performing factor analysis.

### Statistical methods

Data were analyzed using SAS, version 9.4 (SAS Institute, Cary, NC). Results were expressed as numbers and percentages for categorical data. The test of significance between two variables was performed using the Kruskal-Wallis test, comparing the mean number of correct responses by subject characteristics. P-values of ≤ 0.05 were considered statistically significant.

## Results

### Demographic data

There was an unequal distribution of participants by gender with more male respondents (Table 1). The majority of the participants ranged in age between 23 and 25 years (67.6%) and had level A or level B Grade Point Average (GPA, 80%).

### Principal component analysis

Each of the four factors was given a title according to the item’s relationship, as follows: Factor 1, “Knowledge of CLP management,“ with 4 items and 2.02% variance; Factor 2, “Knowledge of dental-related management of CLP,“ with 3 items and 1.93% variance. Factor 3: “Training and exposure to CLP (Interdisciplinary),“ having 5 items and 1.90% variance; and Factor 4, “General knowledge of CLP,“ comprised of 3 items and 1.75% variance (Supplementary File 3a and Supplementary File 3b). Fifteen items were tested for reliability with a Cronbach’s alpha of (α) 0.70 (Supplementary File 3c).

### Characteristics of the participants

The total response rate was 67%, which is considered acceptable [23], and the overall correctness rate was moderate (73.02%, Table 2). A significant gender-related difference was found (P < 0.01), with more correct answers in females than males (Tables 1 and 3). No age-related effects were noted (P > 0.05, Table 1). Most of the participants had level A and level B GPA (39% and 41.9%, respectively). Although no significant difference was found between GPA levels (P = 0.07), interns with higher GPA levels had more correct answers (Tables 1 and 4). There was no significant difference in correct responses based on age or university (Tables 5 and 6). However, variable responses were noticed in relation to the audiology assessment and oronasal fistula in different age groups (Table 5).

**Table 2:** Percentage of correct responses for each domain and question

Main Factors n = 105	Agree n (%)	Disagree n (%)	DNK n (%)	Correct n (%)
**Factor 1: Knowledge of cleft lip and palate management**				
Q3, CLP and Dental anomalies	91 (86.7)	9 (8.6)	5 (4.8)	91 (86.7)
Q6, CLP management and multidisciplinary team	100 (95.2)	1 (1.0)	4 (3.8)	100 (95.2)
Q11, Age of CLP patient and dental implant	75 (71.4)	13 (12.4)	17 (16.2)	75 (71.4)
Q12, CLP and dental occlusion	92 (87.6)	8 (7.6)	5 (4.8)	92 (87.6)
**Factor 2: Knowledge of dental-related management of cleft lip and palate**				
Q7, Pre-surgical orthopedics and surgical outcomes	82 (78.1)	5 (4.8)	18 (17.1)	82 (78.1)
Q10, First-line management of CLP and obturators	87 (82.9)	5 (4.8)	13 (12.4)	87 (82.9)
Q13, Differentiate different types of CLP	82 (78.1)	16 (15.2)	7 (6.7)	82 (78.1)
**Factor 3: Training and exposure to cleft lip and palate (Interdisciplinary)**				
Q5, Phonetics and improper surgery	94 (89.5)	4 (3.8)	7 (6.7)	94 (89.5)
Q8, Importance of audiology assessment	82 (78.1)	12 (11.4)	11 (10.5)	82 (78.1)
Q9, CLP and oronasal fistulae	34 (32.4)	49 (46.7)	22 (21.0)	49 (46.7)
Q14, Exposure to CLP clinical cases	19 (18.1)	82 (78.1)	4 (3.8)	82 (78.1)
Q15, Early diagnosis of CLP	88 (83.8)	10 (9.5)	7 (6.7)	88 (83.8)
**Factor 4: General knowledge of cleft lip and palate**				
Q1, CLP incidence and smoking	28 (26.7)	55 (52.4)	22 (21.0)	28 (26.7)
Q2, CLP incidence and gender	74 (70.5)	15 (14.3)	16 (15.2)	74 (70.5)
Q4, Folic acid decreases risk of CLP	44 (41.9)	18 (17.1)	43 (41.0)	44 (41.9)
**Overall correctness**	/	/	/	**73.02%**
**Range**				(26.7–95.2)

n, number of the participants; Q, question; DNK, Do Not Know

**Table 3:** Correct responses based on gender

	Gender			
Main Factors n = 105	**Total Correct responses** **n = 105** **n (%)**	**Male Correct responses** **n = 57** **n (%)**	**Female** **Correct responses** **n = 48** **n (%)**	**P value**
Factor 1: Knowledge of cleft lip and palate management				
Q3, CLP and Dental anomalies	91 (86.7)	47 (82.5)	44 (91.7)	0.25
Q6, CLP management and multidisciplinary team	100 (95.2)	53 (93.0)	47 (97.9)	0.37
Q11, Age of CLP patient and dental implant	75 (71.4)	38 (66.7)	37 (77.1)	0.28
Q12, CLP and dental occlusion	92 (87.6)	47 (82.5)	45 (93.8)	0.14
Factor 2: Knowledge of dental-related management of cleft lip and palate				
Q7, Pre-surgical orthopedics and surgical outcomes	82 (78.1)	43 (75.4)	39 (81.3)	0.64
Q10, First-line management of CLP and obturators	87 (82.9)	46 (80.7)	41 (85.4)	0.61
Q13, Differentiate different types of CLP	82 (78.1)	41 (71.9)	41 (85.4)	0.11
Factor 3: Training and exposure to cleft lip and palate				
Q5, Phonetics and improper surgery	94 (89.5)	51 (89.5)	43 (89.6)	1.00
Q8, Importance of audiology assessment	82 (78.1)	46 (80.7)	36 (75.0)	0.49
Q9, CLP and oronasal fistulae	49 (46.7)	18 (31.6)	31 (64.6)	< 0.01**
Q14, Exposure to CLP clinical cases	82 (78.1)	38 (66.7)	44 (91.7)	< 0.01**
Q15, Early diagnosis of CLP	88 (83.8)	46 (80.7)	42 (87.5)	0.43
Factor 4: General knowledge of cleft lip and palate				
Q1, CLP incidence and smoking	28 (26.7)	10 (17.5)	18 (37.5)	0.03*
Q2, CLP incidence and gender	74 (70.5)	39 (68.4)	35 (72.9)	0.67
Q4, Folic acid decreases risk of CLP	44 (41.9)	21 (36.8)	23 (47.9)	0.32

n, number of the participants; Q, question; * Significant (P < 0.05); ** Highly Significant (P < 0.01)

**Table 4:** Correct responses based on Grade Point Average (GPA)

	GPA			
Main Factors n = 105	**A** **Correct responses n = 41** **n (%)**	**B** **Correct responses n = 44** **n (%)**	**C/D** **Correct responses n = 20** **n (%)**	**P value**
Factor 1: Knowledge of cleft lip and palate management				
Q3, CLP and Dental anomalies	36 (87.8)	38 (86.4)	17 (85.0)	1.00
Q6, CLP management and multidisciplinary team	40 (97.6)	41 (93.2)	19 (95.0)	0.84
Q11, Age of CLP patient and dental implant	29 (70.7)	35 (79.5)	11 (55.0)	0.15
Q12, CLP and dental occlusion	35 (85.4)	38 (86.4)	19 (95.0)	0.63
Factor 2: Knowledge of dental-related management of cleft lip and palate				
Q7, Pre-surgical orthopedics and surgical outcomes	32 (78.0)	35 (79.5)	15 (75.0)	0.95
Q10, First-line management of CLP and obturators	37 (90.2)	34 (77.3)	16 (80.0)	0.26
Q13, Differentiate different types of CLP	35 (85.4)	32 (72.7)	15 (75.0)	0.36
Factor 3: Training and exposure to cleft lip and palate				
Q5, Phonetics and improper surgery	38 (92.7)	40 (90.9)	16 (80.0)	0.37
Q8, Importance of audiology assessment	31 (75.6)	35 (79.5)	16 (80.0)	0.95
Q9, CLP and oronasal fistulae	27 (65.9)	15 (34.1)	7 (35.0)	< 0.01**
Q14, Exposure to CLP clinical cases	35 (85.4)	36 (81.8)	11 (55.0)	0.03*
Q15, Early diagnosis of CLP	35 (85.4)	39 (88.6)	14 (70.0)	0.17
Factor 4: General knowledge of cleft lip and palate				
Q1, CLP incidence and smoking	13 (31.7)	11 (25.0)	4 (20.0)	0.63
Q2, CLP incidence and gender	31 (75.6)	30 (68.2)	13 (65.0)	0.64
Q4, Folic acid decreases risk of CLP	20 (48.8)	15 (34.1)	9 (45.0)	0.37

n, number of the participants, Q, question; * Significant (P < 0.05); ** Highly Significant (P < 0.01)

**Table 5:** Correct responses based on age

	Age			
Main Factors n = 105	**Total Correct responses** **n = 105** **n (%)**	**Age 23–25 Correct responses n = 71** **n(%)**	**Age 26–30** **Correct responses n = 34** **n(%)**	**P value**
Factor 1: Knowledge of cleft lip and palate management				
Q3, CLP and Dental anomalies	91 (86.7)	59 (83.1)	32 (94.1)	0.22
Q6, CLP management and multidisciplinary team	100 (95.2)	66 (93.0)	34 (100.0)	0.17
Q11, Age of CLP patient and dental implant	75 (71.4)	51 (71.8)	24 (70.6)	1.00
Q12, CLP and dental occlusion	92 (87.6)	60 (84.5)	32 (94.1)	0.22
Factor 2: Knowledge of dental-related management of cleft lip and palate				
Q7, Pre-surgical orthopedics and surgical outcomes	82 (78.1)	58 (81.7)	24 (70.6)	0.22
Q10, First-line management of CLP and obturators	87 (82.9)	58 (81.7)	29 (85.3)	0.79
Q13, Differentiate different types of CLP	82 (78.1)	57 (80.3)	25 (73.5)	0.46
Factor 3: Training and exposure to cleft lip and palate				
Q5, Phonetics and improper surgery	94 (89.5)	63 (88.7)	31 (91.2)	1.00
Q8, Importance of audiology assessment	82 (78.1)	51 (71.8)	31 (91.2)	0.03*
Q9, CLP and oronasal fistulae	49 (46.7)	40 (56.3)	9 (26.5)	< 0.01**
Q14, Exposure to CLP clinical cases	82 (78.1)	57 (80.3)	25 (73.5)	0.46
Q15, Early diagnosis of CLP	88 (83.8)	58 (81.7)	30 (88.2)	0.57
Factor 4: General knowledge of cleft lip and palate				
Q1, CLP incidence and smoking	28 (26.7)	19 (26.8)	9 (26.5)	1.00
Q2, CLP incidence and gender	74 (70.5)	51 (71.8)	23 (67.6)	0.66
Q4, Folic acid decreases risk of CLP	44 (41.9)	25 (35.2)	19 (55.9)	0.058

n, number of the participants, Q, question; * Significant (P < 0.05); ** Highly Significant (P < 0.01)

**Table 6:** Correct responses based on university

	University enrolled in			
Main Factors n = 105	KAU Correct responses n = 55 n(%)	Al-Farabi Correct responses n = 16 n(%)	OthersϮ Correct responses n = 34 n(%)	P value
Factor1: Knowledge of cleft lip and palate management				
Q3, CLP and Dental anomalies	48 (87.3)	13 (81.3)	30 (88.2)	0.79
Q6, CLP management and multidisciplinary team	50 (90.9)	16 (100)	34 (100)	0.14
Q11, Age of CLP patient and dental implant	38 (69.1)	12 (75.0)	25 (73.5)	0.87
Q12, CLP and dental occlusion	47 (85.5)	14 (87.5)	31 (91.2)	0.72
Factor 2: Knowledge of dental-related management of cleft lip and palate				
Q7, Pre-surgical orthopedics and surgical outcomes	40 (72.7)	13 (81.3)	29 (85.3)	0.38
Q10, First-line management of CLP and obturators	43 (78.2)	15 (93.8)	29 (85.3)	0.35
Q13, Differentiate different types of CLP	41 (74.5)	15 (93.8)	26 (76.5)	0.29
Factor 3: Training and exposure to cleft lip and palate				
Q5, Phonetics and improper surgery	49 (89.1)	15 (93.8)	30 (88.2)	1.00
Q8, Importance of audiology assessment	45 (81.8)	10 (62.5)	27 (79.4)	0.27
Q9, CLP and oronasal fistulae	24 (43.6)	8 (50.0)	17 (50.0)	0.83
Q14, Exposure to CLP clinical cases	42 (76.4)	11 (68.8)	29 (85.3)	0.36
Q15, Early diagnosis of CLP	45 (81.8)	15 (93.8)	28 (82.4)	0.63
Factor 4: General knowledge of cleft lip and palate				
Q1, CLP incidence and smoking	14 (25.5)	4 (25.0)	10 (29.4)	0.95
Q2, CLP incidence and gender	43 (78.2)	10 (62.5)	21 (61.8)	0.19
Q4, Folic acid decreases risk of CLP	25 (45.5)	3 (18.8)	16 (47.1)	0.11

n, number of the participants, ϮUmm Al-Qura University; Batterjee Medical College; Ibn Sina National College

### Pattern of responses

A high proportion of correct answers were in respect of CLP treatment, including the demand for multidisciplinary input, the effect of unfavorable surgical outcomes on speech, and the impact of CLP on dental occlusion and anomalies (95%, 89.5%, 87.6%, and 86.7%, respectively, Table 2).

Questions pertaining to dental management such as obturators and pre-surgical orthopedics were correctly answered by the majority of the participants. Most of the participants were able to differentiate between different types of CLP (78.1%). Moreover, most of the participants (78.1%) had no actual encounters with CLP patients. The correct minimum age for the placement of implants in CLP was identified by 71.4% of the participants (Table 2).

The association between CLP and smoking was identified by 26.7% of participants. Similarly, there was limited understanding of the link between oronasal fistulae and CLP, with 32% correctly identifying this (Table 2). The effect of folic acid supplements on reducing orofacial clefting risk was identified by less than half of the participants (41.9%, Table 2).

No gender difference was found in the correctness of answers except for three questions (1, 9, and 14), with females providing more correct answers than males (p = 0.03, p < 0.01, p < 0.01, respectively; Table 3). For questions 9 and 14, there was a substantial positive correlation between GPA level and correct responses (p < 0.01, p = 0.03, respectively), with the largest proportion of correct answers occurring at the A-level (65.9% and 85.4%, respectively, Table 4).

## Discussion

In order to evaluate the knowledge of CLP among recently-graduated dentists we developed a novel questionnaire adhering to standard methodology for questionnaire development [24–26]. Four distinct phases were undertaken, including questionnaire construction, validity and reliability, factor construction and assessment, and questionnaire distribution for target respondents. A good level of internal consistency (Cronbach’s alpha = 0.70) suggests that the 15-item questionnaire is adequate to test the knowledge assessment of dental interns [24]. The overall findings demonstrated gaps in basic understanding and exposure to CLP. There is limited appreciation among dental interns of the risk factors for CLP as well as post-surgical complications. Half of the participants were not aware of the oronasal fistula, which is the most common complication of cleft palate repair due to the failure of wound healing.

The number of correct answers was not associated with the participant’s age or affiliation. Although a non-significant difference was found between low and high GPAs, the number of participants with a C or D grade was half that of those with grades A and B. This suggests that the low overall correctness rate is a function of the structure and delivery of CLP teaching provided by dental schools rather than reflecting students’ ability.

Gender difference in learning capacity has been reported in the literature, including differences in learning methods, habits, and environments [27]. In the current study, female participants had significantly higher knowledge than male in particular questions including prevalence of CLP and associated oronasal complication, which is more linked to theoretical knowledge. Students’ performance could be influenced by the type of teaching methods. Females may perform better in theoretical elements than male [27, 28].

Hypodontia and microdontia are commonly observed in CLP with ectopic eruption, supernumerary teeth and macrodontia less prevalent [29]. In the current study, a high percentage of interns were aware of the association between dental anomalies and CLP. This may relate to the logical perception of the proximity of dentition to the cleft defect.

The need of multidisciplinary team for CLP management was identified by almost all the participants. However, the role of each specialty was not addressed. Therefore, various aspects of CLP management and additional teaching material such as efficient referral and quality of life assessment for both parents and child should be explored in more detail [30]. Case-based learning is considered more effective than other teaching methods in addressing inter-disciplinary care in an enjoyable and interactive way [31]. This approach may therefore be considered in order to produce improved knowledge related to the CLP management.

Patients with CLP suffer from a variety of health complications such as speech and hearing problems, feeding difficulties, and dental complications due to anatomical abnormalities [32–35]. Dental interns awareness of CLP complications was high, with a minor percentage unaware of the importance of effective surgery and audiology assessment and their role in improving speech and hearing. In comparison, a significant proportion of medical students (25%) were aware of the association between speech problems and CLP [36]. More recently, Palee et al. [37] suggested implementation of new education methods such as digital educational games in order to improve CLP knowledge for medical students. However, a systematic review found unclear evidence regarding educational games as a teaching strategy for medical students [38]. Alternatively, a study involving 25 graduates of communication sciences and disorders program, who had not undergone previous CLP training courses, demonstrated the usefulness of video training tutorials for the assessment and treatment of patients with CLP [18].

In regards to post-surgical complications, less than half of our participants were not aware of oro-nasal fistulae in CLP. This is probably correlated to several factors including lack of subject interest, limited teaching material and absence of student assessment for CLP.

A low percentage of dental interns attended CLP clinical sessions in their undergraduate program with 78% not being exposed to CLP cases. This is consistent with the findings of Vallino et al. [36] study, where only (13.9%) of participants had previous exposure to CLP. Chairside teaching sessions would help dental students and/or interns to develop and retain their obtained knowledge and to reshape their education [39, 40]. In addition, the role of the tutors in the clinic and their impact on the learning and teaching process should be highlighted [41]. Therefore, evidence-based chairside teaching is recommended to promote critical thinking development for dental students [42].

Although the literature demonstrates a clear association between maternal smoking and CLP [43], only a quarter of the participants in the current study were aware of this relationship. Gender differences in the prevalence of CLP have been reported in the literature with males being more frequently affected [7]. In the present study, two-thirds of the participants were aware of the differences in CLP incidence by gender. There is also convincing evidence demonstrating that folic acid deficiency in pregnant women may predispose to CLP with a daily intake of 0.4 milligrams of folic acid during pregnancy reducing the risk of having babies with CLP by one-third [44]. In the current study, the importance of folic acid intake for pregnant women in reducing the risk of CLP was recognized by less than 50% of our sample. A study by Alnujaim et al. [45], involving 310 pregnant women, found approximately 50% of the women were aware of the risk of folic acid deficiency and its relation to CLP. However, the internet was found to be the major source of knowledge (34.8%) with health professionals (8.7%) and public health campaigns (8.1%) less influential [46].

The strengths of the current study include the original analysis and the use of a reliable and validated bespoke questionnaire to address the study question. The complete validated questionnaire is provided in supplementary file 2.

In terms of limitations, the generalizability of the findings was limited by the characteristics of the study participants. The limited sample size of 105 participants could impact the generalizability of the findings. In addition, the study was conducted in a specific geographical area (Jeddah, Saudi Arabia), and we acknowledge the potential impact of this localized context on the wider applicability of the findings. Other counties with differing healthcare systems and educational structures may yield different results. Future research involving a more extensive and diverse population of recently graduated dentists may therefore be beneficial in augmenting the insights gained in the present study.

The nature of the study design and the restricted sample comprising dental interns make the study prone to selection bias. The results should therefore be interpreted with caution. Nevertheless, the potential for non-response bias was limited in the present study given the acceptable response rate [23]. In addition, social bias was limited by considering both genders, while questions that might lead to socially desirable responses were avoided.

The pivotal role of oral health care among the multidisciplinary pathways of CLP management was identified. Addressing fundamental knowledge gaps could positively influence patient care and lead to improved outcomes in the context of cleft lip and palate treatment. In particular, enhanced and more focused chairside teaching for dental interns could attenuate the knowledge gap concerning some aspects of CLP. In addition, the role of dentists in the parental education of children with CLP could be considered in more depth.

## Conclusion

A validated questionnaire to explore dental interns’ knowledge of CLP was developed. This was harnessed to highlight areas requiring additional focus, including the etiology and clinical presentation associated with CLP. Given that general dentists are instrumental in the counseling and management of patients with cleft lip and palate, this information can be used to develop bespoke teaching of cleft lip and palate. Dental schools should reflect on the delivery of CLP teaching, in particular, augmenting theoretical content with complementary practical sessions.

### Electronic supplementary material

Below is the link to the electronic supplementary material.

**Supplementary File 1**. 47-item preliminary questionnaire

**Supplementary File 2**. Informed consent and a 15-item questionnaire with correct answers

**Supplementary File 3**a. Eigenvalues of the Correlation Matrix. **Supplementary File 3**b. Rotated Factor Pattern. **Supplementary File 3**c. Internal consistency for reliability (Cronbach alpha)

## Data Availability

The data used to support the findings are available from the corresponding author upon request.

## Abbreviations

CLP: Cleft lip and palate
CL: Cleft lip
CP: Cleft palate
GPA: Grade Point Average
I-CVI: Item Content Validity Index
MSA: Measure of Sampling Adequacy
SD: Standard Deviation
n: Number
DNK: Do Not Know
Q: Question
