# Supplementary material for: Knowledge assessment on cleft lip and palate among recently graduated dentists: a cross-sectional study
Source: BMC Oral Health. 2023 Sep 25;23:689. doi: 10.1186/s12903-023-03388-y (PMC10521468; doi:10.1186/s12903-023-03388-y)
Supplement: Supplementary file 1 — 47-item preliminary questionnaire [file 12903_2023_3388_MOESM1_ESM.docx]

**Manuscript Title:** Knowledge Assessment on Cleft Lip and Palate among Recently Graduated Dentists: A Cross-Sectional Study

**Supplementary File 1.** 47-item preliminary questionnaire

1. **The first section of the questionnaire focuses on your background information.**

- **E-mail: ________________________**
- Male
- Female
- **Age**
- 23–25
- 26–28
- 28–30
- **Current study program**
- Dentistry
- Medicine
- **Grade Point Average (GPA):**
- A
- B
- C
- D
- **University currently enrolled in:**
- King Abdulaziz University
- Umm Al-Qura University
- Ibn Sina National College
- Al-Farabi Private College
- Batterjee Medical College
- Others ______________________.

**2) The following questions will ask about your general knowledge of cleft lip and palate:**

| **Do you agree or disagree with each of the following statements?** | **Agree** | **Disagree** | **Do not Know** |
| --- | --- | --- | --- |
| 1. The incidence of cleft lip and palate is high in Saudi Arabia. | x |  |  |
| 2. The incidence of cleft lip and palate is related to smoking. | x |  |  |
| 3. The incidence of cleft lip and palate is related to gender. | x |  |  |
| 4. Cleft lip and palate is related to intelligence. |  | x |  |
| 5. Cleft lip and palate affects hearing. | x |  |  |
| 6. Cleft lip and palate affects psychological status. | x |  |  |
| 7. Cleft lip and palate is related to lisping. |  | x |  |
| 8. The carrier of the gene responsible for cleft lip and palate shows manifestations of this anomaly. |  | x |  |
| 9. Cleft lip and palate causes dental anomalies. | x |  |  |
| 10. Cleft palate is always associated with a cleft lip. |  | x |  |
| 11. Cleft lip and palate is always present as part of syndromes. |  | x |  |
| 12. Folic acid supplementation reduces the risk of cleft lip and palate. | x |  |  |
| 13. Submucous cleft palate is the most severe form of cleft. |  | x |  |

**3) The next questions will ask about your knowledge of cleft lip and palate management:**

| **Do you agree or disagree with each of the following statements?** | **Agree** | **Disagree** | **Do not Know** |
| --- | --- | --- | --- |
| 14. Everyone with cleft lip and palate needs to have surgery. | x |  |  |
| 15. Cleft lip and palate repair improves speech when it's done at an early age. | x |  |  |
| 16. Phonetics are affected if surgery is not performed correctly. | x |  |  |
| 17. Keeping the wound clean and using ointment improves aesthetic outcomes. |  | x |  |
| 18. Follow-up is required for a better prognosis. | x |  |  |
| 19. The management of cleft lip and palate requires a multidisciplinary team. | x |  |  |
| 20. Dentistry specialists should be involved in the management of cleft lip and palate. | x |  |  |
| 21. There are various methods for cleft lip and palate surgical repair. | x |  |  |
| 22. Pre-surgical infant orthopedics improves surgical outcomes. | x |  |  |
| 23. Pre-surgical infant orthopedics improves nasal projection. | x |  |  |
| 24. Audiology assessment is important in the care of patients with cleft lip and palate. | x |  |  |
| 25. Oronasal fistulae are rarely seen in patients with cleft lip and palate. |  | x |  |
| 26. Early orthodontic care is not important in managing patients with cleft lip and palate. |  | x |  |
| 27. Alveolar bone graft surgery is not indicated in cases with a cleft of the soft palate only. | x |  |  |
| 28. Cleft lip and palate can be detected before birth using prenatal ultrasound. | x |  |  |

**4) The next questions will ask about your knowledge of dental-related management of cleft lip and palate:**

| **Do you agree or disagree with each of the following statements?** | **Agree** | **Disagree** | **Do not know** |
| --- | --- | --- | --- |
| 29. Feeding instructions and obturators are the first line of management for cleft lip and palate patients. | x |  |  |
| 30. A high lip line is a dental complication that may happen after surgery. | x |  |  |
| 31. Dentists are the specialists involved in the construction of the palatal obturators. | x |  |  |
| 32. The minimum age to do dental implants in patients with cleft lip and palate should be above 18 years. | x |  |  |
| 33. Space maintainers are contraindicated as a dental treatment for patients with cleft lip and palate. |  | x |  |
| 34. Cleft lip and palate affects dental occlusion | x |  |  |
| 35. Behavior management is an important aspect of treating children with cleft lip and palate. | x |  |  |
| 36. Maxillary lateral incisors are the most affected teeth in patients with cleft lip and palate. | x |  |  |
| 37. The best way to manage a badly decayed tooth in the cleft area is extraction. |  | x |  |

**5) The final section will ask about your training and exposure to cleft lip and palate:**

| **Please answer the following questions:** | **Yes** | **No** | **Do not know** |
| --- | --- | --- | --- |
| 38. During your academic training, have you had any courses or lectures covering the topic of cleft lip and palate surgery? | x |  |  |
| 39. Have you ever been exposed to cases of cleft lip and/or cleft palate during your clinical training? |  | x |  |
| 40. Are you able to differentiate between the different types of cleft lip and/or palate? | x |  |  |
| 41. Have you ever attended management sessions of cleft lip and/or cleft palate? |  | x |  |
| 42. Are you aware of the speech outcomes after cleft lip and palate repair? | x |  |  |
| 43. Are you comfortable evaluating and managing patients with cleft lip and palate after graduating from dental school? |  | x |  |
| 44. Would it be beneficial to diagnose and treat the cleft lip and palate cases early? | x |  |  |
| 45. Are you aware of the methods of referring a cleft lip and/or cleft palate patient to other specialties? | x |  |  |
| 46. Are you aware of specialized centers to refer patients with cleft lip and palate in your city? | x |  |  |
| 47. Is there any research being done regarding cleft lip and palate in Saudi Arabia? | x |  |  |
