# Supplementary material for: Knowledge assessment on cleft lip and palate among recently graduated dentists: a cross-sectional study
Source: BMC Oral Health. 2023 Sep 25;23:689. doi: 10.1186/s12903-023-03388-y (PMC10521468; doi:10.1186/s12903-023-03388-y)
Supplement: Supplementary file 2 — Informed consent and a 15-item questionnaire with correct answers [file 12903_2023_3388_MOESM2_ESM.docx]

**Manuscript Title:** Knowledge Assessment on Cleft Lip and Palate among Recently Graduated Dentists: A Cross-sectional Study

**Supplementary File 2.** Informed consent and a 15-item questionnaire with correct answers

1. **Informed consent section**

The Perception of Dental Interns towards Cleft Lip and Palate

Dear respected intern,

You are invited to participate in a research study about a dental Intern’s knowledge of cleft lip and palate (CLP). The goal of this research study is to investigate the perception of dental interns towards cleft lip and palate diagnosis and management. This study is being conducted by the following research team: (Dr. Agha, Dr. Helal, Dr. Khafaji, Dr. Farie, Dr. Basri and Dr. Fleming).

Participation in this study is voluntary and anonymous. If you agree to participate in this study, you will need to complete a 15-item questionnaire. It will take about 10 minutes. Participating in this study may not benefit you directly, but it will help us improve the curriculum of dental schools in Jeddah.

You may not be able to skip any question, but you may end the survey at any time. If you agree to participate in this study, the collected information will be kept completely confidential to the full extent of the law. Your information will be assigned a code number that is unique to this study.

If you have further questions, please contact the principal investigators (Dr. Agha and Dr. Helal) on this emails: bahn.agha@uomustansiriyah.edu.iq, nhilal@Kau.edu.sa

By completing this survey, you are consenting to participate in this study.

Note: Interns who are in the Medicine Program are not eligible for the current study. The

questionnaire will be excluded if it is answered by medical interns.

1. **Questionnaire section**

Please answer the following:

**1) Demographic data**

**E-mail: ______________________________________**

1. **Gender:**

- Male
- Female

1. **Age**

- 23–25
- 26–28
- 28–30

1. **Current study program**

- Dentistry
- Medicine

1. **Grade Point Average (GPA):**

- A
- B
- C
- D

1. **University currently enrolled in:**

- King Abdulaziz University
- Umm Al-Qura University
- Ibn Sina National College
- Al-Farabi Private College
- Batterjee Medical College
- Others ______________________.

**2) 15-item questionnaire**

**a. General knowledge of cleft lip and palate**

| **Do you agree or disagree with each of the following statements?** | Agree | Disagree | Do not Know |
| --- | --- | --- | --- |
| The incidence of cleft lip and palate is related to smoking. | x |  |  |
| The incidence of cleft lip and palate is related to gender. | x |  |  |
| Folic acid supplementation reduces the risk of cleft lip and palate. | x |  |  |

**b. Knowledge of cleft lip and palate management**

| **Do you agree or disagree with each of the following statements?** | Agree | Disagree | Do not Know |
| --- | --- | --- | --- |
| Cleft lip and palate cause dental anomalies. | x |  |  |
| The management of cleft lip and palate require a multidisciplinary team. | x |  |  |
| The minimum age to do dental implants in patients with cleft lip and palate should be above 18 years. | x |  |  |
| Cleft lip and palate affect dental occlusion. | x |  |  |

**c. Knowledge of dental-related management of cleft lip and palate**

| **Do you agree or disagree with each of the following statements?** | Agree | Disagree | Do not know |
| --- | --- | --- | --- |
| Pre-surgical infant orthopedics improves surgical outcomes, such as nasal projection, and minimizes lip scarring. | x |  |  |
| Feeding instructions and obturators are the first line of management for cleft lip and palate patients. | x |  |  |
| Are you able to differentiate between the different types of cleft lip and/or palate? | x |  |  |

**d. Training and exposure to cleft lip and palate (Interdisciplinary)**

| **Please answer the following questions:** | Yes | No | Do not know |
| --- | --- | --- | --- |
| Phonetics are affected if surgery is not performed correctly. | x |  |  |
| Audiology assessment is important in the care of patients with cleft lip and palate. | x |  |  |
| Oronasal fistulae are rarely seen in patients with cleft lip and palate. |  | x |  |
| Have you ever attended a clinical session for cleft lip and/or cleft palate. |  | x |  |
| Would it be beneficial to diagnose and treat the cleft lip and palate cases early? | x |  |  |
