# Supplementary material for: Knowledge assessment on cleft lip and palate among recently graduated dentists: a cross-sectional study
Source: BMC Oral Health. 2023 Sep 25;23:689. doi: 10.1186/s12903-023-03388-y (PMC10521468; doi:10.1186/s12903-023-03388-y)
Supplement: Supplementary file 3 — a. Eigenvalues of the Correlation Matrix. Supplementary File 3b. Rotated Factor Pattern. Supplementary File 3c. Internal consistency for reliability (Cronbach alpha) [file 12903_2023_3388_MOESM3_ESM.docx]

**Manuscript Title:** Knowledge Assessment on Cleft Lip and Palate among Recently Graduated Dentists: A Cross-sectional Study

**Supplementary File 3**

**Supplementary File 3a.** Eigenvalues of the Correlation Matrix

|  | Eigenvalue | | Difference | | Proportion | | Cumulative | |
| --- | --- | --- | --- | --- | --- | --- | --- | --- |
| 1 | **3.02917169** | 1.37627473 | | 0.2019 | | 0.2019 | |  |
| 2 | **1.65289696** | 0.09440862 | | 0.1102 | | 0.3121 | |  |
| 3 | **1.55848834** | 0.19867670 | | 0.1039 | | 0.4160 | |  |
| 4 | **1.35981164** | 0.37777294 | | 0.0907 | | 0.5067 | |  |
| 5 | 0.98203870 | 0.03827939 | | 0.0655 | | 0.5722 | |  |
| 6 | 0.94375931 | 0.06267087 | | 0.0629 | | 0.6351 | |  |
| 7 | 0.88108844 | 0.03147315 | | 0.0587 | | 0.6938 | |  |
| 8 | 0.84961529 | 0.15836199 | | 0.0566 | | 0.7505 | |  |
| 9 | 0.69125329 | 0.01783500 | | 0.0461 | | 0.7965 | |  |
| 10 | 0.67341829 | 0.04044638 | | 0.0449 | | 0.8414 | |  |
| 11 | 0.63297191 | 0.13518282 | | 0.0422 | | 0.8836 | |  |
| 12 | 0.49778910 | 0.04426671 | | 0.0332 | | 0.9168 | |  |
| 13 | 0.45352239 | 0.04021246 | | 0.0302 | | 0.9471 | |  |
| 14 | 0.41330993 | 0.03244521 | | 0.0276 | | 0.9746 | |  |
| 15 | 0.38086472 |  | | 0.0254 | | 1.0000 | |  |

Eigenvalues above 1 suggested 4 factors: Total= 15 Average = 1, Overall MSA = 0.672

**Supplementary File 3b.** Rotated Factor Pattern

**1.** Variance Explained by all the Factors

|  | | Factor 1 | | Factor 2 | | Factor 3 | | Factor 4 | |
| --- | --- | --- | --- | --- | --- | --- | --- | --- | --- |
| Q1 | 0.16724 | | -0.05066 | | 0.07581 | | **0.70026** | |  |
| Q2 | -0.07154 | | 0.08644 | | 0.10614 | | **0.58556** | |  |
| Q3 | **0.65760** | | 0.03400 | | 0.09925 | | 0.12938 | |  |
| Q4 | 0.18493 | | 0.11125 | | -0.18008 | | **0.72885** | |  |
| Q5 | 0.23422 | | -0.05171 | | **0.61881** | | -0.03856 | |  |
| Q6 | **0.72834** | | 0.29218 | | 0.08623 | | 0.04625 | |  |
| Q7 | 0.02164 | | **0.73270** | | 0.05544 | | 0.05582 | |  |
| Q8 | -0.20498 | | 0.22352 | | **0.48834** | | 0.44205 | |  |
| Q9 | 0.01423 | | 0.26981 | | **0.51470** | | -0.35147 | |  |
| Q10 | 0.14010 | | **0.60440** | | 0.40193 | | 0.17657 | |  |
| Q11 | **0.48200** | | 0.37200 | | 0.02616 | | 0.03336 | |  |
| Q12 | **0.78462** | | -0.08998 | | 0.10869 | | -0.00849 | |  |
| Q13 | 0.14005 | | **0.79177** | | -0.04225 | | 0.00697 | |  |
| Q14 | 0.03916 | | -0.05178 | | **0.68940** | | 0.04620 | |  |
| Q15 | 0.08477 | | 0.12178 | | **0.54127** | | 0.08756 | |  |

| 2. Variance explained by each factor: | | | |
| --- | --- | --- | --- |
| Factor 1 | **Factor 2** | **Factor 3** | **Factor 4** |
| 2.0236593 | 1.9274096 | 1.9001925 | 1.7491072 |

**Supplementary File 3c.** Internal consistency for reliability (Cronbach alpha)

| **n=105** | **Number of items** | **Cronbach alpha** | **Reliability** |
| --- | --- | --- | --- |
| **Factor1: Knowledge of CLP management**, 4 items (3, 6, 11, 12) | 4 | 0.657 | Questionable |
| **Factor2**: **Knowledge of dental-related management of CLP**, 3 items (7, 10, 13) | 3 | 0.644 | Questionable |
| **Factor3: Training and exposure to CLP (Interdisciplinary)**, 5 items (5, 8, 9, 14, 15) | 5 | 0.544 | Poor |
| **Factor 4: General knowledge of CLP,** 3 items (1, 2,4) | 3 | 0.532 | Poor |
| **Overall** | 15 | **0.700** | **Acceptable** |
